# Epidemiological study of risk factors from nursery and growing–finishing pig farms associated with rate ratio of pleuritis at slaughterhouse in the state of Santa Catarina, Brazil

**DOI:** 10.1007/s11259-026-11101-x

**Published:** 2026-02-24

**Authors:** Nabila Campregher Zaghlout, David Emilio S. N. Barcellos, Karine Ludwig Takeuti, Gustavo Souza e Silva, Mariana Bertolini, Ricardo Yuiti Nagae, Taís Regina Michaelsen Cê, Mariana Santiago, Pâmela Zanatta dos Santos, Ana Paula Gonçalves Mellagi, Fernando Pandolfo Bortolozzo, Rafael da Rosa Ulguim

**Affiliations:** 1 Setor de Suínos, Faculdade de Veterinária, Universidade Federal do Rio Grande do Sul, Porto Alegre, Rio Grande do Sul Brazil; 2 Universidade Feevale, Novo Hamburgo, Rio Grande do Sul Brazil; 3 Iowa State University, Ames, IA United States of America; 4 Setor de Patologia Veterinária, Faculdade de Veterinária, Universidade Federal do Rio Grande do Sul, Porto Alegre, Rio Grande do Sul Brazil; 5 Seara Alimentos Ltda, Itajaí, Santa Catarina Brazil

**Keywords:** Pleurisy, Respiratory disease, Slaughterhouse, Swine

## Abstract

This study aimed to identify risk factors related to management practices, environmental conditions, and infrastructure characteristics in nursery and growing–finishing pig farms located in the state of Santa Catarina, Brazil, and their association with the rate ratio of pleuritis at slaughter. A prospective observational study was conducted using a structured survey on 159 growing–finishing farms, totaling 162,252 slaughtered pigs, of which 5,126 (3.16%) carcasses showed macroscopic pleuritis lesions. Respiratory clinical signs were recorded for each monitored batch, and the characteristics of all nurseries and growing–finishing farms were evaluated. Macroscopic examination at slaughter was performed on all animals presenting pleuritis, assessing the type of exudate, lesion location, and extent, after carcasses were diverted for further inspection. A subsample of 697 pleural swabs and lung fragments was collected for molecular and histopathological analysis. A generalized regression model with a negative binomial distribution was used to estimate rate ratios and build a multivariate model using pleuritis cases as the outcome variable. Histopathological and molecular analyses revealed chronic lesions, and 11.5% of the samples tested positive for at least one pathogen. The findings suggest that pleuritis observed at slaughter likely originates during the early stages of production, when lesion severity may be underestimated and clinical signs overlooked. Moreover, management factors related to health practices, water quality, and the commingling of animals in nurseries and growing–finishing facilities were identified as key contributors to the occurrence and higher prevalence of pleuritis at slaughter.

**Supplementary Information:**

The online version contains supplementary material available at 10.1007/s11259-026-11101-x.

## Introduction

Pleuritis is defined as inflammation of the lungs affecting the pleural membranes, serous surfaces, and chest cavity, which impairs normal lung function. It poses a significant challenge to the swine industry due to its major health impact, in addition to its economic and welfare impacts, particularly in growing–finishing pigs (Pagot et al. 2007). Although its economic effects are not well documented, pleuritis is known to be associated with poor performance, increased mortality, and higher rates of carcass condemnation (Sorensen et al. 2006; Malcher et al. 2024). It also creates problems at slaughterhouses during trimming, leading to additional labor, slower production lines, and increased costs and waste for the industry. Studies from different regions report considerable variability in pleuritis prevalence in commercial pig production systems, ranging from 12% to 41% in European farms and from 5.2% to 9.8% in Norwegian slaughterhouses, depending on the inspection method and health status of the herds (Jäger et al. 2012; Romano et al. 2025). In Brazil, reported pleuritis prevalence is generally lower, ranging from 0.90% to 14,5% across different production systems and regions (Silva et al. 2006; Galdeano et al. 2019; Baraldi et al. 2019; Petri et al. 2023; Arruda et al. 2024; Mengatto et al. 2025).

The clinical presentation of pleuritis is not solely linked to infectious agents; it is a multifactorial condition that can result from various pathogens and be influenced by extrinsic factors such as environmental conditions and management practices (Barcellos et al. 2008; Opriessnig et al. 2011). Both infectious pathogens and non-infectious factors—such as housing, management, infection pressure, and overall herd health—contribute to the prevalence and severity of respiratory diseases in pig populations (Maes et al. 2001). In this context, slaughterhouse data demonstrate strong associations between pleuritic and pulmonary lesions and herd-level management and biosecurity practices (Ruggeri et al. 2020).

The most important risk factors for pleuritis identified in previous studies are associated with the transmission of infectious agents at both the herd and pig levels. These include high pig density in neighboring farms (Cleveland-Nielsen et al. 2002), poor biosecurity (Maes et al. 2001), large herd size or increased number of pigs per pen (Mousing et al. 1990), lack of complete all-in/all-out management (Fraile et al. 2010), and the mixing of pigs during the growing–finishing phase (Cleveland-Nielsen et al. 2002). Pathogen load is also influenced by non-infectious environmental factors that shape the pig’s immune response according to the level of microbial exposure. As a result, the disease outcome depends on the balance between infection pressure and the pig’s capacity to withstand the challenge (Gonyou et al. 2006).

Despite the multifactorial nature of pleuritis, most studies have focused primarily on infectious agents, with limited integration of management practices, environmental conditions, and structural characteristics at the farm level. Previous studies have assessed pleuritis together with other respiratory lesions or under substantially different conditions, such as full-cycle herds, PRRS-positive populations, laboratory-based diagnostics, or binary farm-level outcomes (Stärk 2000; Maes et al., 2001; Merialdi et al. 2012; Fablet et al. 2012; Ruggeri et al. 2020). Therefore, there is a need to investigate pleuritis as a primary outcome in growing–finishing pigs from PRRS-free and multi-site production systems. Thus, the aim of this study was to evaluate the relationship of management practices, environmental conditions, and the structural characteristics of nurseries and growing–finishing multi-site pig production system with the rate ratio of pleuritis in slaughtered pigs in southern Brazil.

## Materials and methods

### Farms, animals, and housing conditions

The study was conducted during the fall (March–May) and winter (June–August) seasons on nursery and growing–finishing pig farms located in the western region of Santa Catarina, Brazil in a system with a known history of respiratory diseases. Pig production in this region is characterized by vertically integrated systems, with a high density of commercial farms operating under standardized management, nutrition, and health protocols. Production is based on multi-site systems, in which nursery and growing–finishing phases are segregated from each other and from sow farms, and animals are raised under intensive systems, with high levels of biosecurity and technological input. All farms were endemic for influenza A virus and *Mycoplasma hyopneumoniae*, while some nurseries and growing–finishing farms were free from *Actinobacillus pleuropneumoniae*. Sows were vaccinated according to the company’s established immunization protocol, which included vaccines against reproductive diseases and atrophic rhinitis, as well as enteric vaccines administered to ensure passive immunity of piglets via colostrum, specifically against *Escherichia coli* and *Clostridium perfringens* type A. Piglets were vaccinated against *Mycoplasma hyopneumoniae* and additionally received autogenous vaccines targeting *Pasteurella multocida* and *Glaesserella parasuis*. Nursery and growing–finishing barns were equipped with curtains to control temperature and ventilation. During the nursery and growing–finishing phases, pigs were fed exclusively dry diets, provided either in pelleted or mash form. The pigs, all belonging to the same company, were raised under varying management and environmental conditions across the nursery and growing-finishing farms. All animals were slaughtered at a single federally inspected slaughterhouse under veterinary supervision.

### Study design

An observational prospective study was carried out on distinct 159 growing–finishing pig farms. Prior to slaughter analysis, seven company-trained veterinarians assessed the farms using a structured survey designed to standardize data collection and ensure consistent interpretation of the questions. The variables included in the farm survey were selected based on a combination of previously reported risk factors for respiratory disease and pleuritis, established epidemiological principles, and the practical knowledge of veterinarians and researchers in intensive pig production systems. Management-related variables were chosen due to their known influence on animal mixing, infection pressure, and stress, while environmental and structural factors were included based on their role in modulating pathogen survival, exposure, and the pig immune response. Although not all variables were derived from individual studies, their inclusion was biologically plausible and consistent with factors commonly evaluated in respiratory disease investigations in swine production systems (Stärk 2000; Maes et al. 2001b; Barcellos et al. 2008; Opriessnig et al. 2011). An overview of the investigated factors is presented in Table 1S. During slaughter, all animals showing pleuritis were identified through macroscopic examination, and a random subsample of these cases was selected for laboratory diagnostic testing.

### Pleural lesion scoring, macroscopic evaluation, and sampling

Each batch of growing–finishing pigs (*n* = 159), comprising a total of 162,252 slaughtered animals, was examined at the slaughterhouse during routine inspection. All carcasses were individually evaluated by the official veterinary inspection service. Carcasses presenting macroscopic lesions were diverted to final inspection and subsequently assessed by the research team for detailed characterization of pleural lesions. In total, 5,126 carcasses exhibiting pleuritis and diverted to final inspection were macroscopically examined and classified according to the type of exudate as fibrous, fibrinous, suppurative, granulomatous, or fibrin–hemorrhagic (Zachary and McGavin 2012). Additionally, pleuritis lesions were classified by location and extent using the Slaughterhouse Pleurisy Evaluation System (SPES) described by Dottori et al. (2007). Only carcasses with macroscopic lesions and SPES scores of 2 to 4 were diverted to the final inspection. Carcasses classified as SPES score of 0 or 1—without macroscopic lesions suggestive of condemnation—were not diverted. In accordance with current Brazilian legislation (Brasil 1995), carcasses were classified by the official veterinarian for one of the following three outcomes: released without restrictions; conditional use after removal of thoracic organs and parietal pleura, for products such as salted meats, cooked sausages, preserves, or lard; or total condemnation for rendering into meat-and-bone meal and grease.

A subsample of 0 to 5 animals per batch was randomly selected for laboratory analysis, resulting in 697 lung samples. Pleural swabs and lung fragments were collected from the affected areas for molecular and histopathological examination, respectively. Pleural swabs (ABSORVE^®^, Brazil) were refrigerated at 4 °C and processed within 24 h for molecular testing.

### DNA extraction and polymerase chain reaction (PCR)

DNA extraction from all samples (*n* = 697) was performed using a commercial DNA extraction kit (QuantiNova PCR Kit, QIAGEN, USA) according to the manufacturer’s instructions, with processing carried out using the MagMax system. The extracted DNA was subsequently analyzed by quantitative PCR. Pleural swab samples were analyzed for *Actinobacillus pleuropneumoniae* (Tobias et al. 2012), *Glaesserella parasuis* (Turni et al. 2010), *Pasteurella multocida* (Devi et al. 2018), and *Mycoplasma hyorhinis* (Tocqueville et al. 2014).

### Histological analysis

Of the 697 samples collected, 59 were selected for histopathological examination. Selection was based on molecular results—specifically, samples that tested PCR-positive for at least one of the pathogens investigated in this study. Lung fragments (approximately 8 cm^3^) were taken from visibly affected regions, fixed in 10% buffered formalin, and subsequently processed for histopathological evaluation (Allen 1992). Pleural and lung samples, containing transition areas between regions with and without macroscopic lesions were collected. Each fragment was embedded in paraffin, sectioned, and stained with hematoxylin and eosin (Allen 1992). Samples were considered indicative of *P. multocida* infection when the pleura was thickened with a fibrin layer, abundant neutrophils or degenerated inflammatory cells, and necrotic debris (Oliveira Filho et al. 2015). Samples showing necrohemorrhagic consolidation along with diffuse, moderate acute fibrinous pleuritis—characterized by accumulation of fibrinous exudate on the pleural surface—were classified as suspected *A. pleuropneumoniae* infections (Arenales et al. 2022). Lesions suspected of *G. parasuis* infection were identified by diffuse thickening of alveolar septa and marked infiltration of neutrophils in alveolar and bronchiolar lumen (Arenales et al. 2022), while *M. hyorhinis* lesions were characterized by bronchial and/or bronchiolar epithelial damage, including intraepithelial microabscesses, inflammation and necrosis, and bronchial-associated lymphoid tissue (BALT) hyperplasia (Lin et al. 2006).

### Statistical analysis

The observational unit was the batch of animals from each growing–finishing farm, with one batch per farm included in the study. A multivariable regression model with a negative binomial distribution was built, using the number of pleuritis cases as the numerator and the total number of slaughtered animals per batch as the denominator, to identify factors associated with pleuritis rate ratio. First, univariate analyses were performed between each explanatory variable (Table 1S) and the outcome. This step aimed to identify variables associated with pleuritis rate ratio; only those with *P* ≤ 0.20 were eligible for inclusion in the initial multivariable model (step two). The multivariable model was then constructed using a manual stepwise forward selection procedure. Variables with *P* ≤ 0.20 in the univariate analysis were included one at a time, and those with *P* > 0.05 were excluded one at a time until the final model included only variables with *P* ≤ 0.05. The Akaike Information Criterion (AIC) value was used to compare model fit after each exclusion, with the lowest AIC value indicating the best fit (Akaike 1974). Potential confounders and interactions were evaluated based on biological plausibility and relevant literature. Multicollinearity among predictors was assessed in the final model using the variance inflation factor (VIF), and variables with VIF > 5 were excluded. Variables not included in the initial multivariable model due to *P* > 0.20 in the univariate analysis were reintroduced individually into the final model to verify whether they remained non-significant in the presence of potential confounders (Dohoo et al. 2003).

A separate set of models was developed to assess the impact of pleuritis prevalence on key performance indicators. A linear regression model was used to examine the relationship between pleuritis prevalence and mortality during the growing–finishing phase, as well as slaughter weight. The frequency distributions of SPES scores, macroscopic findings, and histopathological characteristics were analyzed using the chi-square test and in this case the animal was used as the observational unit. Descriptive data for herd size and key production indicators were expressed as mean ± SD. All analyses were performed using R statistical software (R Core Team 2023).

## Results

The mean number of pigs housed and slaughtered per growing–finishing farm was 1,072.69 ± 846.21 and 1,020.45 ± 812.21, respectively. A detailed description of the overall production performance of the growing–finishing farms is provided in Table 1. The average prevalence of pleuritis per batch was 3.1% (ranging from 0.0% to 12.2%), regardless of lesion severity (Table 1).

**Table 1:** Descriptive production performance of 159 growing–finishing farms and the occurrence of pleuritis observed at slaughter

Variable	Mean ± SD	Median [Min–Max]
Housed animals per farm (n)	1072.69 ± 846.21	780 [250–5040]
Slaughtered animals per farm (n)	1020.45 ± 812.21	741 [227–4939]
Weight (kg)		
Start of housing	28.35 ± 4.12	27.75 [17.68–44.14]
Slaughter	128.42 ± 4.38	128.58 [115.77–141.78]
ADG (kg/d)	0.958 ± 0.06	0.962 [0.769–1.235]
Mortality (%)	1.93 ± 1.20	1.63 [0–6.90]
Total slaughtered pigs (n)	162.252	-
Total pigs with pleuritis* (n)	5.126	-
Total pigs with pleuritis per farm (n)	32.24 ± 34.08	20 [0–198]
Pigs with pleuritis per farm (%)	3.07 ± 2.51	2.50 [0–12.17]

ADG, average daily gain; SD, standard deviation

*Only pigs with a Slaughterhouse Pleurisy Evaluation System score of ≥ 2 (Dotori et al., 2007) were considered

## Univariate analyses

In total, 38 variables (Table 1S) were included in the univariate analysis to assess their association with pleuritis. Among these, 11 variables were significantly associated with pleuritis at slaughter (*P* ≤ 0.05): final weight at nursery, weaning age, number of weaning sources, number of nursery sources, respiratory clinical signs in the growing–finishing phase, percentage of pigs medicated parenterally in the growing–finishing phase, water source, mass water medication, observation of dyspnea, and finishing mortality and mortality due respiratory causes (Table 1S).

### Multivariable analysis

Of the 38 variables analyzed in the univariate step, 19 met the inclusion criterion (*P* ≤ 0.20) for the final multivariable regression model (Table 2). The final model identified several risk factors for pleuritis from the weaning to the growing–finishing phases (Table 3). Batches with more than five weaning sources had a 1.85-fold higher rate ratio (RR) for pleuritis compared with batches originating from a single source (*P* < 0.01). For each additional day of housing in the nursery, the RR increased by 1.06 times (*P* = 0.01). Conversely, higher final body weight at the nursery reduced the RR for pleuritis by 0.95 times (*P* < 0.01). The use of water from a closed source reduced the RR for pleuritis by 0.75 times (*P* = 0.02) compared with an open water source. By contrast, the presence of water blades (water-filled area within the pen) inside pens increased the RR by 1.36 times (*P* = 0.02). Furthermore, in growing–finishing batches showing clinical signs of porcine respiratory disease complex (cough and/or dyspnea) and requiring mass water medication, the RR for pleuritis was 1.94 times higher than in batches without porcine respiratory disease complex or medication (*P* = 0.01).

**Table 2:** Variables identified in the univariate analysis of facilities, management, and production parameters associated with the rate ratios (RR) of pleuritis in slaughtered pigs

Variable	Type	RR	95% CI	*P*-value
*Facilities*				
Floor type	Categorical			
Compact		Ref.		
Slatted		0.81	[0.62–1.07]	0.13
Curtain condition	Categorical			
New		Ref.		
Old		0.94	[0.66–1.34]	0.75
With malfunction		1.26	[0.91–1.73]	0.15
Animals per water nipple		1.06	[0.99–1.12]	0.08
Water blade present	Categorical			
No		Ref.		
Yes		1.27	[0.97–1.67]	0.09
Water source	Categorical			
Opened		Ref.		
Closed		0.72	[0.55–0.95]	0.02
*Management and production parameters*				
Weaning age (days)	Categorical			
21		Ref		
21 and 28		1.62	[1.17–2.30]	< 0.01
28		0.78	[0.59–1.03]	0.07
Number of weaning sources *	Categorical			
1		Ref		
2–4		1.34	[0.95–1.87]	0.09
5–13		2.01	[1.39–2.87]	< 0.01
Number of nursery sources *	Categorical			
Single		Ref.		
Multiple		0.64	[0.48–0.84]	< 0.01
Average number of days in nursery	Continuous	1.04	[0.99–1.08]	0.06
Final weight at nursery (kg)	Continuous	0.94	[0.91–0.97]	< 0.01
Fallback finishing pigs (%)	Continuous	1.02	[1.00–1.05]	0.07
Respiratory pathogens in finishing pigs	Categorical			
No		Ref.		
Yes		1.28	[0.99–1.67]	0.06
Clinical signs of PRDC in finishing pigs	Categorical			
No		Ref		
Yes		0.72	[0.56–0.92]	< 0.01
Dyspnea observed	Categorical			
Yes		Ref.		
No		1.32	[1.01–1.70]	0.04
Percentage of medicated pigs	Continuous	1.00	[1.00–1.01]	0.01
Mass water medication	Categorical			
No		Ref		
Yes		0.74	[0.57–0.96]	0.02
Reintroduction of recovered pigs	Categorical			
No		Ref.		
Yes		1.27	[0.99–1.64]	0.06
Finishing mortality (%)	Continuous	1.16	[1.04–1.29]	< 0.01
Respiratory finishing mortality (%)	Continuous	1.26	[1.10–1.45]	< 0.01

RR, rate ratio; Ref., reference; CI, confidence interval; PRDC, porcine respiratory disease complex represented by clinical signs of cough and/or dyspnea; Water blade, water-filled area within the pen to define the dirty area used for defecation and urination; Water source, open indicates surface water sources; closed indicates deep water sources; Smudge, accumulation of dirt and organic material on the pen floor

*Each batch of pigs in the growing–finishing phase was tracked to identify the numbers of weaning and nursery sources

**Table 3:** Final multivariable model of factors associated with the rate ratios (RR) of pleuritis in slaughtered pigs

Variables	RR	95% CI	*P*-value
Intercept	0.00	[0.00–0.03]	< 0.01
Number of weaning sources			
1	Ref.		
2–4	1.29	[0.93–1.76]	0.12
5–13	1.85	[1.33–2.54]	< 0.01
Average number of days in nursery	1.06	[1.02–1.10]	0.01
Nursery final weight	0.95	[0.92–0.97]	< 0.01
Water source in growing–finishing phase			
Open	Ref.		
Closed	0.75	[0.59–0.96]	0.02
Water blade present			
No	Ref.		
Yes	1.32	[1.03–1.69]	0.02
Clinical signs of PRDC			
No	Ref.		
Yes	0.92	[0.69–1.24]	0.58
Mass water medication			
No	Ref.		
Yes	0.82	[0.58–1.19]	0.28
Interaction			
	Ref.		
Clinical signs of PRDC in finishing pigs [yes] × Mass water medication [yes]	1.94	[1.19–3.14]	0.01

RR, rate ratio; Ref., reference; CI, confidence interval; PRDC, porcine respiratory disease complex represented by clinical signs of cough and/or dyspnea; Water blade, water-filled area within the pen to define the dirty area used for defecation and urination; Water source, open indicates surface water sources; closed indicates deep water sources; Smudge, accumulation of dirt and organic material on the pen floor

### Impact of pleuritis on productive performance during the growing–finishing phase

In terms of performance indicators on growing–finishing farms, an increase in the prevalence of pleuritis within batches was associated with higher mortality rates (*P* ≤ 0.01) and a reduction in slaughter weight (*P* = 0.04) (Table 4).

**Table 4:** Production performance indicators of growing–finishing farms in relation to prevalence of pleuritis at slaughter

Variable	Estimate	95% CI	*P*-value
General mortality (%)	0.09	[0.02–0.17]	0.01
Respiratory-related mortality (%)	0.10	[0.04–0.15]	< 0.01
Pig slaughter weight (kg)	−0.28	[− 0.55 to − 0.00]	0.04

CI, confidence interval

### Characterization of lesions and laboratory assays

**Fig. 1:**
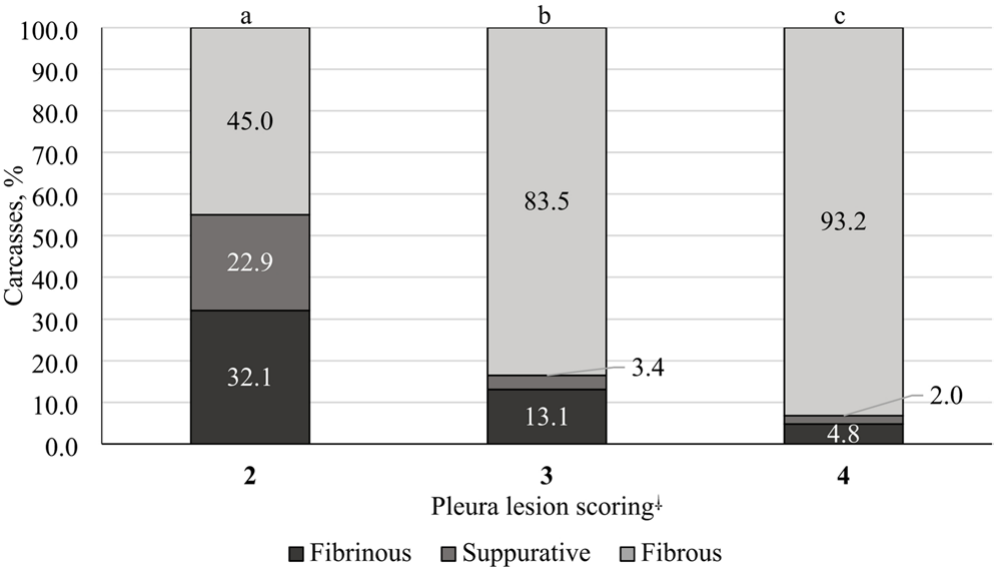
Relationship between the macroscopic exudates of pleuritis lesions and pleural lesion scores in pig carcasses (*P* < 0.01) ^⸸^Pleural lesion scores were described by Dottori et al. (2007). In total, 5,110 carcasses were included in the comparison. Carcasses with fibrin–hemorrhagic lesions were observed in only 16 cases (score 2, *n* = 13; score 3, *n* = 1; score 4, *n* = 2) and were therefore excluded from this analysis

Among the 5,126 pigs diagnosed with pleuritis, 13.6%, 37.4%, and 49.0% presented SPES scores of 2, 3, and 4, respectively. Based on macroscopic assessment, 82.9% of pleuritis cases were classified as fibrous, 11.5% as fibrinous, 5.3% as suppurative, and 0.3% as fibrin–hemorrhagic. Significant differences were observed among all SPES scores regarding the macroscopic characteristics of lesions (*P* < 0.01). Acute lesions, represented by the sum of fibrinous and suppurative exudates, were most frequent in pigs with SPES scores of 2 (55%) (Fig. 1). By contrast, chronic lesions characterized by fibrous exudate predominated in pigs with SPES scores of 3 (83.5%) and 4 (93.2%) (Fig. 1).

Of the 697 samples analyzed by PCR, 11.5% were tested positive for at least one pathogen targeted in this study. *Pasteurella multocida* (detected alone or in combination with *A. pleuropneumoniae*) was the most prevalent pathogen (Table 5). The exudate in *P. multocida*-positive lungs was predominantly fibrous (51.1%), followed by suppurative (25.5%), fibrinous (19.1%), and fibrin–hemorrhagic (4.3%). The second most common pathogen detected was *G. parasuis* (either alone or in combination with *A. pleuropneumoniae* and *M. hyorhinis*), with lesions classified as fibrous (57.9%), fibrinous (31.6%), and suppurative (10.5%).

**Table 5:** Frequency of PCR-positive samples among 697 pleural samples collected

Pathogens	Positives/total positives, *n*/*n* (%)	Positives/total samples, *n*/*n* (%)
*Actinobacillus pleuropneumoniae*	5/80 (6.25)	5/697 (0.70)
*Actinobacillus pleuropneumoniae + Glaesserella parasuis*	1/80 (1.25)	1/697 (0.16)
*Actinobacillus pleuropneumoniae + Pasteurella multocida*	1/80 (1.25)	1/697 (0.16)
*Glaesserella parasuis*	17/80 (21.25)	17/697 (2.40)
*Glaesserella parasuis + Mycoplasma hyorhinis*	1/80 (1.25)	1/697 (0.16)
*Mycoplasma hyorhinis*	9/80 (11.25)	9/697 (1.30)
*Pasteurella multocida*	46/80 (57.50)	46/697 (6.60)
Total	80/80 (100)	80/697(11.5)

Histopathological analysis (*n* = 59) revealed a high proportion of lesions suggestive of *P. multocida* infection (42.4%), followed by *G. parasuis* (32.2%). Microscopic evaluation also confirmed that most lesions were chronic (84.7%), regardless of SPES classification (Table 6). The predominant histopathological finding in slaughtered pigs was fibrous pleuritis, and chronic lesions were observed in 50 of 59 samples.

**Table 6:** Frequency of histopathological characteristics in 59 pleural and lung samples collected at slaughter, according to pleural lesion score (SPES)

Histopathological description of pleural lesions	Pleural lesion score, *n*/*n* (%)*			
	2	3	4	Total
Chronic	12/16 (75.0)	18/22 (81.80)	20/21 (95.24)	50/59 (84.75)
Subacute	2/16 (12.5)	2/22 (9.10)	1/21 (4.76)	5/59 (8.47)
Acute	2/16 (12.5)	2/22 (9.10)	0/21 (0.00)	4/59 (6.78)
Total	16/59 (27.12)	22/59 (37.29)	21/59 (35.59)	59/59 (100)

No differences were found between histopathological characteristics of pleural lesions and lesion scores (*P* = 0.45)

SPES, Slaughterhouse Pleurisy Evaluation System

*Described by Dottori et al. (2007)

Regarding carcass disposition, among the 5,126 carcasses with pleuritis, 84.7% were released without restriction; 9.7% were designated for conditional use, partial condemnation, or preservation; and 5.6% were totally condemned.

## Discussion

In this observational study, 159 growing–finishing farms were analyzed, comprising a total of 162,252 slaughtered pigs. Among these, 5,126 carcasses (3.16%) presented macroscopic lesions of pleuritis, a proportion consistent with rates reported in previous studies conducted in Brazil (Baraldi et al. 2019; Galdeano et al. 2019). Most of the pleurisy lesions observed were chronic in nature, and the study highlights several factors in the nursery and growing–finishing phases that were associated with the rate ratio of pleuritis. This response observed in a multi-site system corroborates findings from farrow-to-finish farms reported by Fablet et al. (2012); Maes et al. (2001b), who also suggested that herd characteristics indirectly influence infection pressure through management practices, including those originating during early production stages.

Certain management strategies related to nursery housing appear to act as predisposing factors for pleuritis. In particular, commingling piglets in nurseries was associated with higher pleuritis occurrence at slaughter. When more than five different nursery sources were combined within a single growing–finishing unit, the risk of pleuritis increased by 1.85 times compared with batches originating from a single nursery source. This effect may be explained by the introduction of pigs with differing sanitary statuses, facility types, and management practices, which heightens the infectious challenge and promotes the circulation of multiple pathogens and strains. In addition, moving and mixing piglets can increase stress and negatively affect immune function (Salak-Johnson and McGlone 2007; Jäger et al. 2012).

Another important finding from the multivariable analysis was the influence of the drinking water source on pleuritis rate. In this study, the use of a closed water source in growing–finishing farms had a protective effect, as these farms showed a 25% lower rate ratio of pleuritis at slaughter. Water quality is a critical component of biosecurity in pig production and should be regarded as a potential route for the introduction of bacterial and viral pathogens (Alarcón et al. 2021). The role of ponds and lakes—common examples of open water sources—in harboring respiratory viruses has already been emphasized in the literature. Respiratory pathogens can be transmitted indirectly through contaminated water sources (Rohani et al. 2009). Similarly, the presence of water blades in growing–finishing farms increased the risk of pleuritis at slaughter by 1.32 times. It is plausible that drinking water or water blades become contaminated with respiratory pathogens, which may generate aerosols that pigs can easily inhale during the growing–finishing phase, thereby increasing the risk of pleuritis at slaughter.

In addition, we found that batches of growing–finishing pigs showing respiratory clinical signs and treated with mass water antibiotic therapy had a higher risk of presenting pleuritis at slaughter. It was expected that batches exhibiting coughing would show more lesions at slaughter. Although mass water medication—administered when more than 10% of animals displayed clinical signs—may have alleviated symptoms, it likely did not prevent the subsequent development of pleuritic lesions. Unfortunately, data on the interval between treatment and slaughter were unavailable, preventing a direct association with lesion scores. Nonetheless, the observation that batches requiring mass water medication for respiratory disease showed higher pleuritis rate ratio indicates that this variable serves as a relevant predictor of pleuritis at slaughter.

Kuberka et al. (2024) also examined the relationship between management practices, facility characteristics, and the occurrence of pneumonic lesions in pig farms. They found that the presence of slatted floors was strongly associated with higher pneumonia scores. However, herd size, stocking density, daily temperature variation, and the use of all-in/all-out systems did not significantly influence lung lesion scores. Interestingly, in our study, several variables—including type of floor, curtain quality, number of animals per building, nebulization, cleaning and disinfection protocols, and type of ventilation—were not significantly associated with pleurisy. Consistent with previous research showing an association between observable respiratory disease during the growing–finishing phase and pleuritis at slaughter (Fraile et al. 2010), our results revealed higher average mortality and respiratory-related mortality in batches with increased pleuritis rate ratio. In general, pigs affected by pleuritis exhibit reduced growth rates, and those requiring pleural stripping experience delayed slaughter times (Malcher et al. 2024; Przyborowska et al. 2024). Moreover, because lung lesions can progress and regress over the pigs’ lifespan, they may have largely resolved by slaughter (Meyns et al. 2011), potentially leading to underestimation of herd-level pleurisy prevalence. Therefore, herd diagnosis based solely on slaughter checks may be biased, and slaughter assessments should ideally be complemented by other monitoring methods such as PCR testing.

The results of our study suggest that several explanatory variables related to pleuritis originated during the nursery phase. The duration of the nursery phase and the final body weight at nursery exit are likely to influence pleuritis occurrence indirectly by influencing early-life exposure to respiratory pathogens. Longer nursery periods may increase cumulative exposure time to infectious agents during a critical phase of immune development, potentially facilitating subclinical infections that progress to chronic pleural lesions later detected at slaughter. Similarly, nursery exit weight may reflect differences in health status, management quality, or infection pressure during early production stages. This is supported by the fact that most pleuritis lesions observed at slaughter were fibrous and chronic in nature, as indicated by the exudate classification, histopathology, and SPES scoring. Merialdi et al. (2012) also reported a high prevalence of predominantly chronic pleuritic lesions at slaughter. Interestingly, the prevalence of lungs showing chronic microscopic characteristics and chronic exudate was identical (82.9%) and closely matched the proportion of lungs classified as SPES 3 or 4 (86.4%). This prevalence is higher than that of severe pleurisy reported by Malcher et al. (2024), who used the same scoring system. These findings suggest that the SPES scoring method proposed by Dottori et al. (2007) and the exudate classification described by Zachary and McGavin (2012) are reliable tools for classifying pleuritis lesions in slaughtered pigs, even without histopathological examination. Furthermore, veterinarians working on inspection lines can effectively use these techniques to classify lesions and determine the appropriate destination for carcasses. In line with the histopathological results, our study showed that 84.7% of carcasses with pleuritis were released without restriction, as the lesions were predominantly chronic.

The PCR assay revealed a low number of positive results, with DNA detected in only 11.5% of samples for at least one of the four pathogens tested. This low detection rate may be explained by the fact that infection likely occurred during the early stages of production and had largely resolved by the growing–finishing or slaughter phases, making bacterial DNA more difficult to detect. Although identifying specific pathogens responsible for pleuritis was not the main objective of this study, *A. pleuropneumoniae*, *G. parasuis*, *M. hyorhinis*, and *P. multocida* (serotypes A and D)—either alone or in combination—were the most frequently detected agents by PCR. It is important to note, however, that in qualitative risk assessments, these pathogens are not considered hazardous to human health through pork consumption (Costa et al. 2017).

In line with these findings, another consideration in this study concerns the age at which pathogens of public health importance exert their greatest impact and their viability in the carcass. Chronic pleuritis does not appear to pose a risk to consumers, which supports the possibility of pleura removal without necessarily divert the carcass to the inspection department —an approach already permitted in Brazil following recent regulatory updates. Other studies have also demonstrated the reliability of visual inspection in confirming the absence of viable bacteria in chronic pleural lesions, as well as the absence of respiratory pathogens classified as biological risks within the pork production chain, thereby supporting the unrestricted use of carcasses with chronic pleural lesions (Rocha et al. 2022). Although chronic pleural adhesions (SPES scores 3 and 4) may delay carcass processing due to pleural stripping, these lesions do not necessarily result in sanitary condemnation when no public health risk is identified, and carcasses may be released for human consumption following individual inspection. Taken together, the results of this study indicate that management practices during the nursery and growing–finishing phases, along with structural aspects of growing–finishing facilities, are associated with the rate ratio of pleuritis. These variables should be regarded as risk factors when developing strategies to reduce pleuritis occurrence in swine production systems.

Pleuritis occurrence leads to economic losses both on farms and in slaughterhouses, mainly due to increased carcass processing time and condemnations resulting from acute lesions. Slaughter inspection was performed under routine commercial conditions with standardized line speed and inspection staff. Although subtle lesions may have been missed, most pleuritis cases were moderate to severe (SPES 3–4), and any under detection is unlikely to bias the observed associations. Farm assessments inherently involve some subjectivity; however, the present study used a standardized questionnaire and veterinarian training, which likely minimized observer bias, and the consistency of associations across farms suggests a limited impact on the study results. The wider applicability of the present findings depends on similarities in production systems and sanitary conditions. The associations identified in this study are likely relevant for other intensive and vertically integrated pig multi-site production systems in Brazil and in countries with comparable biosecurity and disease profiles, but may not apply to regions where respiratory pathogen circulation and disease dynamics differ substantially. Future studies should aim to quantify the economic impact of pleuritis in slaughtered pigs and assess whether this impact varies across herds or depends on specific commercial requirements.

## Conclusion

This study suggests that most pleuritis lesions observed in slaughtered pigs were chronic, as indicated by exudate classification, PCR results, and histopathological findings. Management-related factors—such as the number of weaned piglet sources in the nursery, the number of days spent in the nursery, and the final piglet weight at the end of the nursery phase—were associated with pleuritis rate ratio. In addition, structural aspects of the growing–finishing facilities, including the type of water source and the presence of water blades in pens, also contributed to pleuritis risk. Furthermore, the occurrence of respiratory signs and the need for mass water medication during the growing–finishing phase were associated with a higher rate ratio of pleuritis.

## Supplementary Information

Below is the link to the electronic supplementary material.

Supplementary Material 1

## Data Availability

No datasets were generated or analysed during the current study.
