# Supplementary material for: Epidemiological study of risk factors from nursery and growing–finishing pig farms associated with rate ratio of pleuritis at slaughterhouse in the state of Santa Catarina, Brazil
Source: Vet Res Commun. 2026 Feb 24;50(3):169. doi: 10.1007/s11259-026-11101-x (PMC12932352; doi:10.1007/s11259-026-11101-x)
Supplement: Supplementary file 1 — Supplementary Material 1 [file 11259_2026_11101_MOESM1_ESM.docx]

**Table 1S.** Variables included in the farm survey and their significance in the univariate regression analysis of pleuritis incidence in slaughtered growing–finishing pigs

| Variable | Levels (if applicable) | P-value |
| --- | --- | --- |
| *Facilities* |  |  |
| Number of finishing barns per farm | Value | 0.70 |
| Stocking density in finishing phase (m^2^/pig) | Value | 0.81 |
| Cooling system in grow–finishing barns | Yes/No | 0.28 |
| Floor type | Compact/Slatted | 0.13 |
| Pen wall type | Whole/Slatted | 0.66 |
| Curtain condition | New/Slight malfunction/Old | 0.11 |
| Curtain system | Manual/Automatic | 0.53 |
| Feeder material | Wood/Plastic/Steel/Concrete | 0.43 |
| Water source | Open/Closed | 0.02 |
| *Management* |  |  |
| Distance to nearest pig unit (m) | Value | 0.45 |
| Downtime between batches (days) | Value | 0.57 |
| Smudge | Yes/No | 0.83 |
| Cleaning and disinfection | Yes/No | 0.59 |
| Number of pigs per drinker | Value | 0.08 |
| Number of pigs per feeder | Value | 0.31 |
| Detergent type | Alkaline/Neutral/None | 0.97 |
| Disinfectant used for fogging | Quaternary ammonia/Glutaraldehyde/None | 0.49 |
| Water blade present | Yes/No | 0.09 |
| Fogging with disinfectant during the batch | Yes/No | 0.35 |
| Fly control | Yes/No | 0.98 |
| Mass water medication | Yes/No - Reason | 0.02 |
| Sex of finishing pigs | Female/Male/Mixed | 0.25 |
| *Productive parameters* |  |  |
| Number of pigs per batch | Value | 0.68 |
| Weaning age | 21/28 days | <0.01 |
| Weaning sources | Single/2–4/5–13 | <0.01 |
| Weaning weight (kg) | Value | 0.25 |
| Nursery sources | Single/Multiple | <0.01 |
| Nursery weight (kg) | Value | <0.01 |
| Nursery mortality (%) | Value | 0.68 |
| Average number of days in nursery (days) | Value | 0.09 |
| Parenterally medicated pigs (%) | Continuous | 0.01 |
| Dyspnea observed | Yes/No | 0.04 |
| Diagnostic of respiratory pathogens (finishing phase) | Yes/No | 0.06 |
| Reintroduction of recovered pigs | Yes/No | 0.06 |
| Finishing mortality (%) | Value | <0.01 |
| Fallback finishing pigs (%) | Value | 0.07 |
| Finishing respiratory mortality (%) | Value | <0.01 |
| Clinical signs of PRDC in finishing pigs | Yes/No | <0.01 |

PRDC, porcine respiratory disease complex represented by clinical signs of cough and/or dyspnea; Water blade, water-filled area within the pen to define the dirty area used for defecation and urination; Water source, open indicates surface water sources; closed indicates deep water sources; Smudge, accumulation of dirt and organic material on the pen floor;
